# Spatial modeling of biological patterns shows multiscale organization of *Arabidopsis thaliana* heterochromatin

**DOI:** 10.1038/s41598-020-79158-5

**Published:** 2021-01-11

**Authors:** Javier Arpòn, Kaori Sakai, Valérie Gaudin, Philippe Andrey

**Affiliations:** Institut Jean-Pierre Bourgin, INRAE, AgroParisTech, Université Paris-Saclay, 78000 Versailles, France

**Keywords:** Image processing, Statistical methods, Cellular imaging, Nuclear organization

## Abstract

The spatial organization in the cell nucleus is tightly linked to genome functions such as gene regulation. Similarly, specific spatial arrangements of biological components such as macromolecular complexes, organelles and cells are involved in many biological functions. Spatial interactions among elementary components of biological systems define their relative positioning and are key determinants of spatial patterns. However, biological variability and the lack of appropriate spatial statistical methods and models limit our current ability to analyze these interactions. Here, we developed a framework to dissect spatial interactions and organization principles by combining unbiased statistical tests, multiple spatial descriptors and new spatial models. We used plant constitutive heterochromatin as a model system to demonstrate the potential of our framework. Our results challenge the common view of a peripheral organization of chromocenters, showing that chromocenters are arranged along both radial and lateral directions in the nuclear space and obey a multiscale organization with scale-dependent antagonistic effects. The proposed generic framework will be useful to identify determinants of spatial organizations and to question their interplay with biological functions.

## Introduction

From macro-molecular complexes and organelles up to cells and tissues, living systems present complex spatial organizations, with specific arrangements of their elementary components. Deciphering these organizations is a major goal in biology because they are tightly related to biological functions. For example, at the sub-cellular level, the spatial distribution over the plasma membrane of a transporter involved in glucose homeostasis is altered during insulin stimulation and in insulin-resistant context^1^. At the whole-cell level, the clustering of lysosomes is dynamic, coordinates metabolic responses to nutrient availability and determines their interactions with endosomes^2,3^. At a tissular level, the spatial patterning of stomata is involved in leaf physiology, including carbon assimilation and water-use efficiency^4^. The number and size of elementary components in a biological system and the shape of the domains within which these objects are distributed are inherently variable. In addition, spatial organizations are most frequently three-dimensional and the underlying rules are generally impossible to detect visually from observed patterns. These are all major obstacles to the understanding of spatial organization principles.

Spatial interactions are major determinants of spatial organizations. Typically, they manifest themselves by mutual dependency in the positioning of objects, such as attraction (leading to clustering) or repulsion (leading to regular distributions). Spatial statistics offer powerful methods to evaluate the existence and nature of such interactions by providing comparison tests between observed distributions and model distributions based on hypothetical rules of organization^5,6^. These methods have been essentially developed for applications in domains, such as forestry, geography, and epidemiology, where the data consist of single observations, in two dimensions, within arbitrary observation windows and where individual objects are assimilated to points. By contrast, spatial data in biological imaging typically come as series of repeated observations, in three dimensions, within finite domains and are related to objects that cannot be reduced to points. While it is essential to take into account these specific features of biological data for unbiased spatial analyses, they have been rarely considered, and only separately^7–13^. In addition, spatial studies generally rely on the comparison between observed patterns and complete spatial randomness, which represents a basic reference model only. In this work, we address the need for new spatial statistical approaches and for more elaborated reference models to finely dissect spatial organization rules in living systems from imaging data.

We choose the interphase cell nucleus as a unique and challenging model system for investigating spatial patterns and organization rules in biological systems. Indeed, it is a highly-organized and dynamic system that contains numerous structurally, biochemically, epigenetically or topologically defined compartments (reviewed in^14–19^). Intricate links have been established between the spatial and compartmentalized organization of the cell nucleus and gene regulation, developmental processes, and diseases^20,21^. Constitutive heterochromatin, one of the first described nuclear compartments^22,23^, can form well-defined chromocenters (CCs) in several animal and plant species, such as *Arabidopsis thaliana*. The polymorph appearance and dynamics of CCs according to cellular, developmental or physiological contexts and environmental responses^8,19,24–31^ suggest functional roles of CCs. A better characterization of the CC spatial organization is required to evaluate their roles in nuclear organization and genome functions.

Here, we developed a framework to investigate spatial interactions and principles of organization in living systems and used it to reveal new distribution rules for plant CCs. Our framework relies on modeling the spatial distribution of real-sized objects within finite 3D domains from multiple imaging observations. We previously demonstrated the more regular distribution of constitutive heterochromatic compartments than expected under randomness, and the conservation of this specific nuclear feature in animals and plants^32^. We introduced in our new framework a series of models (sets of rules that describe the positioning of objects) that increasingly fit with the observed CC distribution. Our results challenge the classical view according to which interactions with the nuclear periphery are sufficient to account for CC distribution. We show that additional interactions are required to fully explain the spatial arrangement of CCs and highlight a multiscale organization of CCs with maximal repulsion at large scale. The capacity of the proposed generic framework to unmask elaborate organizational rules offers new perspectives for analyzing spatial organizations in cell and developmental biology.

## Testing departure from complete randomness shows a multiscale organization of chromocenters

**Figure 1:**
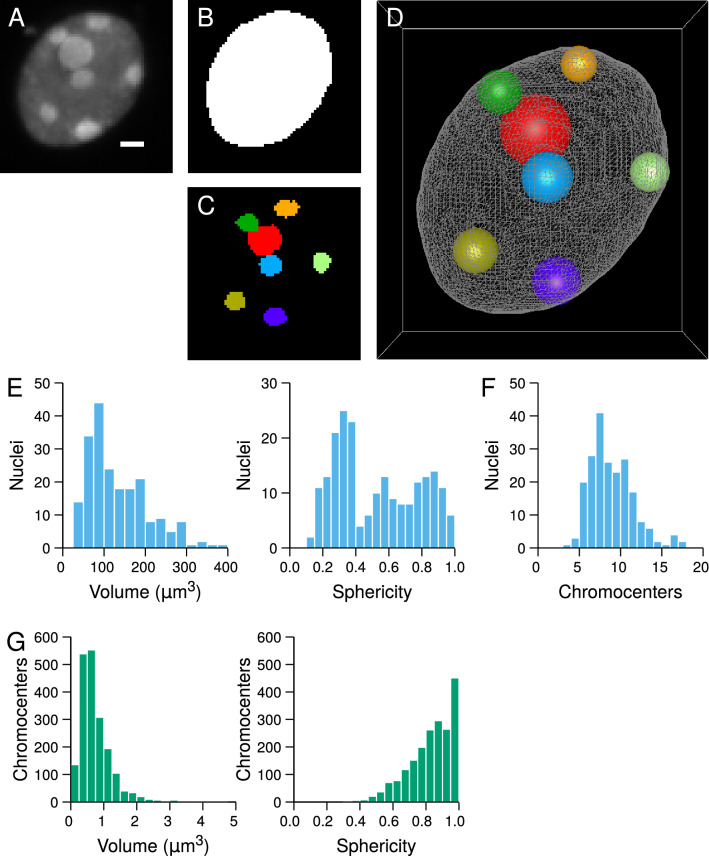
Generation of 3D representations in continuous space from discrete image data and quantitative image analysis of *Arabidopsis thaliana* plantlet nuclei. (**A**) Confocal image of an *A. thaliana* DAPI-stained nucleus. Chromocenters appear as bright foci. Scale bar: 1 $\upmu$m. (**B**) Segmentation mask of the nucleus. (**C**) Segmentation masks of the chromocenters. ABC show maximum intensity projections of the corresponding 3D images. (**D**) 3D reconstruction of the nucleus (triangular mesh) with chromocenters represented by their equivalent spheres. (**E**) Distribution of nuclear size and shape parameters. (**F**) Distribution of the number of chromocenters per nucleus. (**G**) Distribution of chromocenter size and shape parameters.

The framework we developed to investigate rules of organization in 3D domains consists in evaluating the goodness-of-fit between spatial models and sets of observed object patterns. The input data, extracted from collections of segmented images (Fig. 1A–C), are 3D patterns of objects represented in the continuous space by their equivalent spheres and distributed within confined domains represented by their boundaries (Fig. 1D). Each observed pattern is quantitatively characterized using spatial descriptors (distance functions). Using continuous space representations allows to avoid distance measurement approximations that are inherent to discrete image representations we previously used^32^. Patterns simulated according to the model to be tested are quantified with the same spatial descriptors. The adequacy between the observed pattern and the simulated patterns is quantified using normalized measures called Spatial Distribution Indexes (SDI). The goodness-of-fit between a model and a set of observed patterns is tested based on the uniformity of the SDI distribution (see Methods). Indeed, uniform SDI distributions are expected when comparing observed patterns to a model that fits with their organization rules (Figure S1AB). Conversely, non-uniform SDI distributions are expected when the tested spatial model does not fully account for the spatial organization of the analyzed patterns (Figure S1CD and EF).

Domain size and shape, number and sizes of individual objects are confounding factors for the analysis of spatial interactions. For example, the distances between randomly distributed objects depend on the domain size. To ensure differences between observed patterns and model simulations only result from differences in organization rules, these factors are set in the model to observed values and shapes.

To demonstrate its potential, we applied our new framework to 3D patterns of chromocenters (CCs) within isolated nuclei of *Arabidopsis thaliana* seedlings. 3D confocal microscopy images of DAPI-stained nuclei were acquired and processed to extract nuclear boundaries, CC centroid positions and CC volumes (Fig. 1A–D). Measuring nuclear size and shape revealed morphological heterogeneity in the population of analyzed nuclei and showed that many nuclei departed from perfectly spherical shapes (Fig. 1E). The distribution of the number of CC per nucleus was also spread. Many nuclei contained more than 10 CCs (Fig. 1F), showing a large proportion of nuclei had undergo endoreduplication. Chromocenters were also morphologically variable (Fig. 1G). However, in contrast with nuclei, CC variability essentially affected their size, as shown by the large coefficient of variation (CV) of CC volume (CV = 67.9%) compared to CC sphericity (CV = 16.4%). The distribution of CC sphericity was concentrated near the maximal value 1.0, showing they could correctly be assimilated to spherical objects (Fig. 1G). Altogether, nuclear and CC features confirmed that several parameters (domain size, domain shape, number of objects, size of objects) could potentially confound the analysis of CC spatial organization rules, and confirmed the need for a methodology that can take into account these potential biases by appropriate normalization procedures.

**Figure 2:**
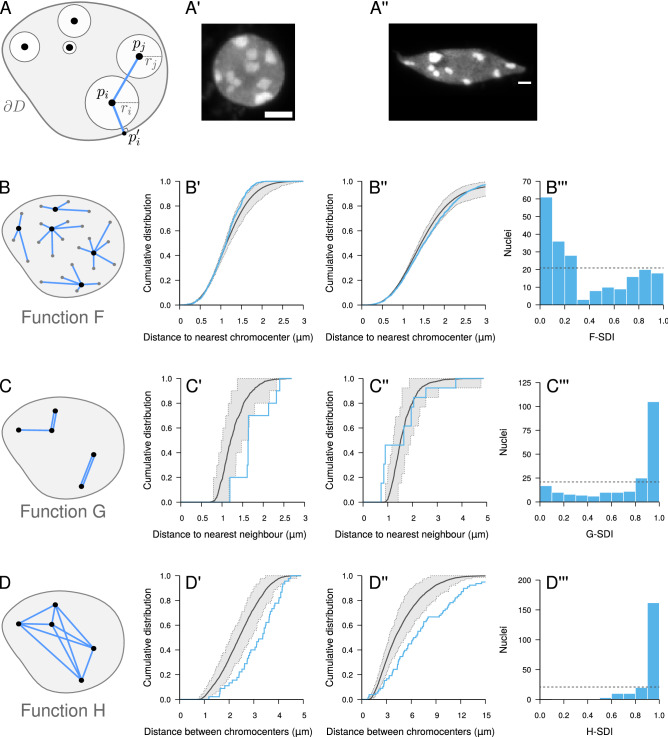
Completely random object distribution model and its application to chromocenters in *A. thaliana* cell nuclei. (**A**) Completely random object model in confined domain. Objects are placed at random positions, avoiding intersections with other objects or with domain boundary $\partial D$. $p_i$: centroid position of object *i*; $r_i$: equivalent radius of object *i*; $p_i'$: boundary point closest to $p_i$. (**A′**, **A”**) Two sample nuclei from the analyzed population (scale bar: 5 $\upmu$m). (**B**–**D**) Distance measurements used to compute functions *F*, *G*, and *H*, respectively. *Black dots*: object centroids; *Gray dots*: space positions. (**B′**, **B”**) Measured (*Blue*) and model (*Black*) functions *F* in the two sample nuclei shown in (**A′**, **A”**). *Dotted lines*: lower and upper limit of the 95% envelope (*Grey*) under the model. (**B’’’**) Distribution of the *F*-SDI in the population of nuclei. *Dotted line*: distribution expected under the completely random model. (**C′**, **C”**, **C’’’**) Same as (**B′**, **B”**, **B’’’**) with function *G*. (**D′**, **D”**, **D’’’**) Same as (**B′**, **B”**, **B’’’**) with function *H*.

Comparing observed patterns to a completely random model of object distribution, we previously showed that CCs in *A. thaliana* cell nuclei exhibit a more regular than random, repulsive-like spatial distribution^32^. This conclusion was based on short-range spatial descriptors (function *F*: cumulative distribution of the distance between a typical 3D position and its closest object; function *G*: cumulative distribution of the distance between each object and its nearest neighbor; Fig. 2B,C) that revealed negative spatial interactions at a local scale. Here, we examined whether such negative interactions were also present at larger scales by using an additional descriptor, function *H* (cumulative distribution of the distance between any pair of objects), which quantifies inter-distances at all scales (Fig. 2D)^5^.

We first tested our new framework using functions *F* and *G*. These functions were computed for each observed pattern and compared to ${\hat{F}}_{\mathrm{rand}}$ and ${\hat{G}}_{\mathrm{rand}}$, the estimates of these functions under the completely random object model (Fig. 2A), which were obtained by averaging over model simulations. For each observed pattern, the corresponding simulations were conditioned on the observed shape of the nucleus and on the number and individual sizes of CCs in the pattern. A similarly conditioned second set of simulations were performed to estimate the expected fluctuations around the averages under the model and to compute the SDIs (see Methods).

For most nuclei (as the one shown in Fig. 2A′), the observed function *F* was shifted to the left of ${\hat{F}}_{\mathrm{rand}}$ (Fig. 2B′), showing that regions devoid of CCs were on average smaller than under a random distribution. The slope of the observed function *F* was steeper, pointing to a more homogeneous size distribution of empty spaces between CCs. Function *G* was shifted to the right of ${\hat{G}}_{\mathrm{rand}}$ (Fig. 2C′), corresponding to larger distances between each CC and its nearest neighbour than under the random model. In some nuclei (as the one shown in Fig. 2A”), the *F* and *G* functions exhibited a reverse behavior (Fig. 2B”,C”), suggesting that some CCs in these nuclei were closer than under complete randomness. At the population level, the bimodal *F*-SDI (Fig. 2B′′′) and *G*-SDI (Fig. 2C′′′) distributions confirmed the mixed trend towards regularity or clustering at the local scale. We found no link between this heterogeneity of SDIs and the heterogeneity in nucleus morphology or number of chromocenters, suggesting no relation with ploidy levels (Figure S2). The majority of *F*-SDI and *G*-SDI values were close to 0 and 1, respectively, showing the prevailing trend towards regularity. These results confirm our previous results showing a more regular than random, repulsive-like organization of CCs^32^.

Function *H* was shifted to the right of ${\hat{H}}_{\mathrm{rand}}$ (Fig. 2D′), suggesting that the negative spatial interactions were manifest up to the largest spatial scale. The few nuclei showing a trend for clustering on a local scale (Fig. 2B”C”) exhibited the same shift of *H* to the right of ${\hat{H}}_{\mathrm{rand}}$ (Fig. 2D”). Hence, for these nuclei, there was a mix of both positive and negative interactions depending on the considered spatial scale. At the population level, the *H*-SDI had a unimodal distribution that was concentrated towards the right end of the SDI range (Fig. 2D′′′). Overall, the multiscale analysis resulting from the combination of functions *F*, *G*, and *H* reveals that (i) the distribution of CCs systematically exhibits regularity at a global scale and that (ii) the negative spatial interactions that subtend this regularity can be partially released or reversed on a local scale.

## New descriptors demonstrate spatial interactions between chromocenters and nucleus boundary

The repulsive-like distribution of CCs led us to examine their radial positioning. The analysis of radial positioning is essential in spatial studies of biological objects because of the functional importance of domain boundaries such as cell membrane or nuclear envelope. This type of analysis is based on distance measurements between objects and domain center or periphery, possibly including normalization scheme for variations in domain size^33^. However, small distances to the border are not indicative of a preferential positioning at the periphery. For example, in a unit sphere, a uniformly random point has a probability of $\sim$.85 of being closer to the border than the center. Hence, evaluating spatial interactions with domain boundary cannot be based on measured distances only but requires appropriate statistical testing.

**Figure 3:**
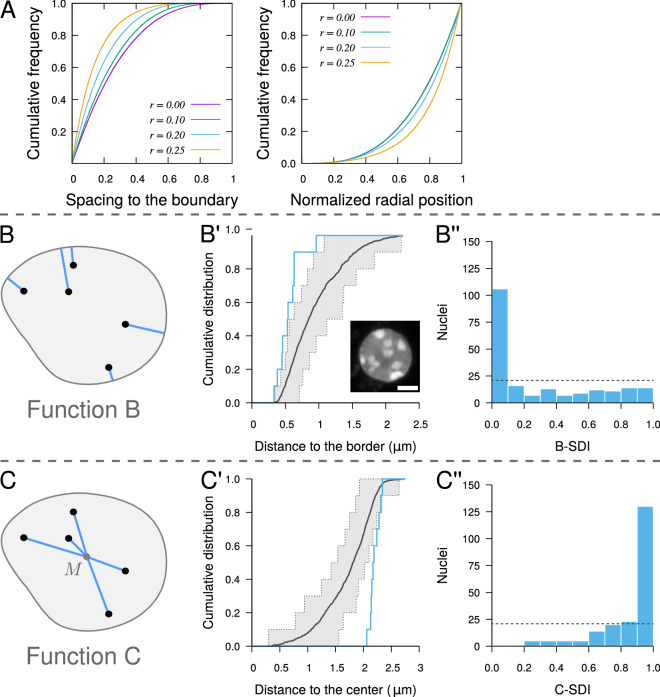
Testing spatial interactions between objects and domain boundary with application to the peripheral positioning of chromocenters in *A. thaliana* cell nuclei. (**A**) Radial positioning of spherical objects uniformly distributed at random within a unit sphere (averages over 10,000 simulated patterns containing 10 objects of radius *r*). *Left*: spacing between objects and sphere boundary. *Right*: distance to sphere center following normalization of maximum distances to 1. (**B**) Distance measurement between object centroid and its closest point on domain boundary used for function *B*. Same notations as in Fig. 2. (**B′**) Function *B* (*Blue*) measured in a sample nucleus (*Inset*; scale bar: 2 $\upmu$m). *Black* and *Grey*: average function and 95% envelope, respectively, under the completely random model. (**B”**) Distribution of the SDI computed using function *B* in the population of nuclei. *Dotted line*: distribution expected under a random organization. (**C**) Distance measurement between object and domain centroids used for function *C*. *M*: domain centroid. (**C′**, **C”**) Same as (**B′**, **B”**) with function *C*.

Because mutual exclusion between real-sized objects can influence their positioning, we first examined the impact of object size on radial distributions. Using the random object model, we simulated ensembles of non-intersecting spheres of varying radius within a spherical domain. We found that radial distributions were depending on object sizes. The spacing to the border was on average smaller for large objects compared with small ones (Fig. 3A, *Left*). Large objects were also relatively closer to the domain boundary than small objects (Fig. 3A, *Right*). This shows that the reduced spacing between large objects and domain boundary was not just a simple consequence of their larger size but was also resulting from mutual exclusion between objects. These results highlight how important it is to take into account object sizes for an unbiased radial analysis.

We introduced two new spatial descriptors to evaluate the peripheral positioning of real-sized objects within confined domains, allowing to test the preferential positioning of objects towards or away from the domain boundary (function *B*) or center (function *C*). Function *B* is the cumulative distribution function of the distance between object centroid and the border of the domain (Fig. 3B). Function *C* is the cumulative distribution function of the distance between object and domain centroids (Fig. 3C). Though they are based on classical distance measurements (distance to domain boundary or center), introducing functions *B* and *C* into our framework allows the unbiased testing of radial and peripheral positioning of objects. When the SDIs are computed using simulations from the completely random model, the null hypothesis in the population test based on function *B* or on function *C* is that object positions are independent from the domain boundary or center, respectively. For each function, the null hypothesis is rejected if the corresponding SDI distribution is not uniform. For example, significantly small or large *B*-SDIs correspond to object distributions with preferential localization close to, or away from the domain boundary, respectively (Figure S1D and F).

For most nuclei, the individual functions *B* exhibited a trend towards smaller distances to the border compared with a completely random distribution of CCs conditioned by actual CC sizes and domain shape (Fig. 3B′). This was confirmed at the population level by the strongly skewed distribution of *B*-SDIs towards 0, which was significantly different from the expected uniform distribution under the random model (Fig. 3B”). However, the *B*-SDI distribution was heterogeneous, with a large peak near 0 followed by a uniform distribution up to 1. A large proportion of nuclei were flat. If CCs are randomly distributed along the direction of flattening but are close to the domain periphery in the plane orthogonal to this direction, function *B* may fail to reveal significant interactions between objects and domain boundary but function *C* should unmask the peripheral location. Consistently, function *C* measured on individual nuclei was generally shifted towards higher distances as compared with the random distribution (Fig. 3C′). This was confirmed at the population level by the skewed distribution towards 1 of the *C*-SDI (Fig. 3C”). Overall, these results provided an unbiased demonstration of the preferential localization of CCs at the periphery of the nucleus in *A. thaliana* cells.

## Orbital model reveals new spatial interactions beyond chromocenter peripheral positioning

Numerical simulations showed that a preferential localization at the periphery of the domain suffices to induce a repulsive-like (more regular than random) organization: in patterns simulated with object attraction to the border, distances to nearest neighbors were larger than under complete randomness, as shown in individual plots of function *G* and in the population distribution of the *G*-SDI (Figure S1C). We thus asked if the spatial heterogeneity (non-homogeneous probability for a CC to be located at some position) demonstrated above in the peripheral analysis was sufficient to explain the discrepancy between observed patterns and complete randomness, or if additional spatial interactions between CCs were also involved.

**Figure 4:**
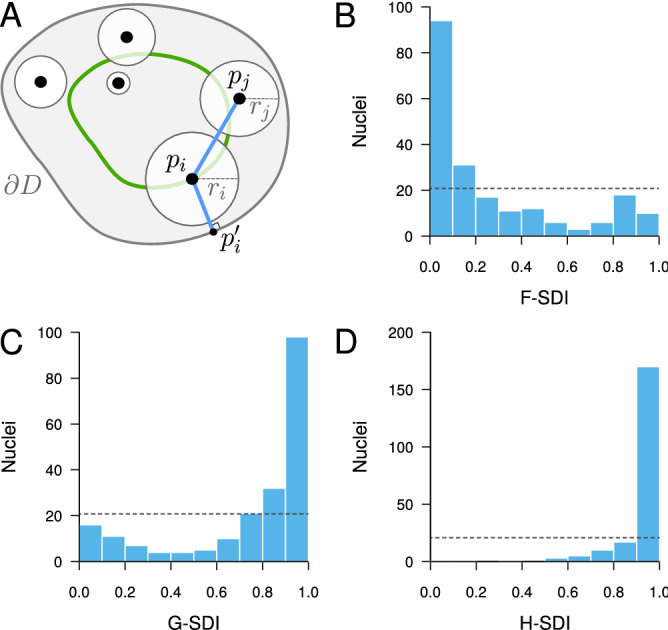
Testing the contribution of spatial heterogeneity (interaction between objects and domain border) to object distributions in confined domains, with application to *A. thaliana* chromocenters. (**A**) Orbital model: object positions are randomly positioned over orbits (*Green*), avoiding overlaps between objects. Same notations as in Fig. 2. (**B**–**D**) Evaluation of the orbital model on the distribution of chromocenters in *A. thaliana* cell nuclei using the SDI method and functions *F* (**B**), *G* (**C**) and *H* (**D**).

We addressed this question by introducing an orbital model of object distribution, generalizing the orbital model of object pair introduced in^34^. In our orbital model, each object is uniformly distributed at random over an orbit defined by a given distance between the object centroid and the domain boundary (Fig. 4A). Different objects can be located on orbits with different distances to the boundary. As in the completely random model, object sizes are taken into account to prevent intersections. The null hypothesis of the population test is that the object distribution is fully determined by the orbital distances. The alternative is that spatial interactions other than the interaction with domain boundary determine the positioning of objects. The test is performed by comparing simulated orbital patterns to the observed ones based on SDI distribution uniformity.

We applied the orbital model to CCs, setting the orbital distance of each CC in model simulations to its measured distance to the nuclear boundary. As above for the comparison to the random model, the other model parameters (nuclear size and shape, number and individual sizes of CCs) were all taken from experimental observations. The three distance functions *F*, *G* and *H* all revealed a marked difference between the observed patterns and model predictions. The *F*-SDI distribution was concentrated near 0 (Fig. 4B) and conversely, the *G*- and *H*-SDI distributions were concentrated near 1 (Fig. 4C,D), all suggesting a more regular (repulsive-like) spatial distribution of CCs as compared with the orbital model. We concluded that the apparent repulsion between CCs (Fig. 2) cannot result only from their preferential positioning at the periphery. Additional, negative spatial interactions (direct or indirect) govern the distribution of CCs in *A. thaliana* nuclei.

## Maximal repulsion accounts for the large scale distribution of chromocenters

Confronting observed CC distributions to the completely random and to the orbital models showed a trend towards regularity that manifests repulsive spatial interactions. This raises the question of the strength of these interactions, and in particular to what extent the apparent repulsion between CCs, and hence the regularity of their distribution, is maximal or not.

**Figure 5:**
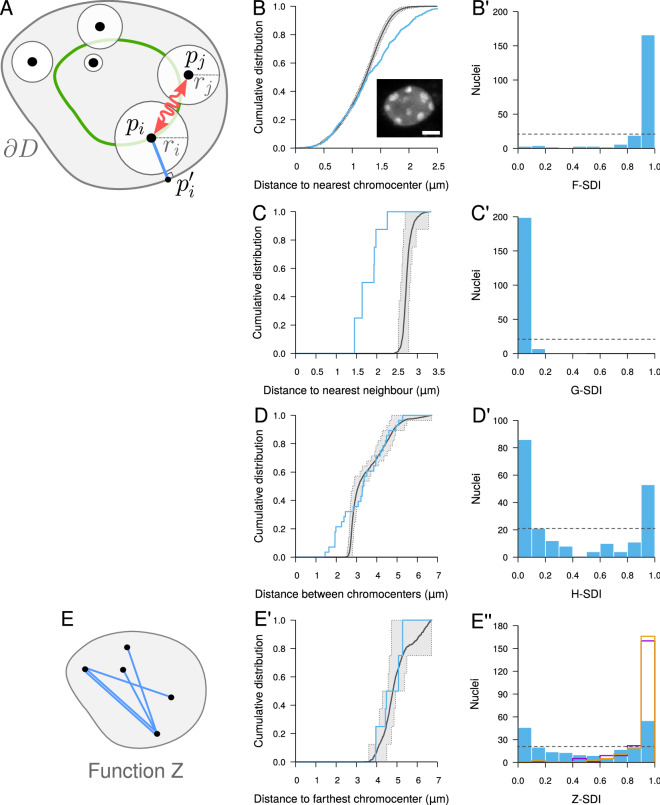
Orbital maximal repulsion model of object distribution and its application to the distribution of chromocenters in *A. thaliana* cell nuclei. (**A**) Orbital maximal repulsion model: objects are positioned as far as possible from their nearest neighbor while moving over their respective orbits. (**B**) Measured (*Blue*) and model (*Black*) functions *F* in a sample nucleus (*Inset*; scale bar: 2 $\upmu$m). *Dotted lines*: lower and upper limit of the 95% envelope (*Grey*) under the model. (**B′**) Distribution of the *F*-SDI in the population of nuclei. *Dotted line*: distribution expected under the orbital maximal repulsion model. (**C**, **D**) Same as (**B**) for functions *G* and *H*. (**C′**, **D′**) Same as (**B′**) with the *G* and *H*-SDIs. (**E**) Distance measurements used to compute function *Z*. (**E′**, **E”**) Same as (**B**, **B′**) for function *Z*. *Magenta* and *Orange*: *Z*-SDI histograms for the completely random and orbital models, respectively.

To address this question, we defined a maximal repulsion model, which corresponds to the distribution in which the average distance between each object and its nearest neighbor is maximized. We combined this model with the orbital model. The resulting orbital maximal repulsion model was obtained from the maximal repulsion model by introducing the additional constraints that objects should be maintained over their orbits (Fig. 5A). The input parameters for the orbital maximal repulsion model are the same as for the orbital model (domain shape, number of objects, individual object sizes and orbital distances). For each individual CC pattern we analyzed, these parameters were set in model simulations to the values measured on the pattern.

At the local scale, as assessed using functions *F* and *G*, there was a strong discrepancy between observed CC patterns and model predictions. For most nuclei, the observed function *F* showed an excess of large distances compared with the model (Fig. 5B). This showed that regions devoid of CCs were larger in the observed patterns than expected under the orbital maximal repulsion model, thus corresponding to less repulsive patterns. Symmetrically, the observed function *G* was almost systematically shifted to the left of the distribution expected under the model, revealing smaller nearest neighbor distances (Fig. 5C). These individual observations were corroborated by the population distribution of the *F*-SDI (Fig. 5B′) and of the *G*-SDI (Fig. 5C′), which were concentrated near 1 and 0, respectively. These results show that spatial interactions between CCs at short range are not maximally repulsive.

For most nuclei, function *H* exhibited a differential behavior depending on the distance between CCs. In the small distance range, there was a strong excess of CC inter-distances as compared with the orbital maximal repulsion model (Fig. 5D), which was consistent with the behavior of function *G*. However, in the intermediate to long distance ranges, the observed function *H* was much closer to the average distribution expected under the model, and for most nuclei it was generally enclosed within the 95% envelope (Fig. 5D). This biphasic behavior of function *H* was reflected at the population level by the bimodality of the *H*-SDI distribution (Fig. 5D′). To examine more closely the patterns in the long distance range, we introduced a new spatial descriptor, function *Z*, defined as the cumulative distribution function of the distance between each object and its farthest neighbor (Fig. 5E). For most nuclei, function *Z* was very close to the distribution expected under the model (Fig. 5E′). At the population level, the *Z*-SDI covered the whole range between 0 and 1 (Fig. 5E”, *Blue*). This widely spread distribution radically differed from the *Z*-SDI distributions obtained with the completely random and orbital models (Fig. 5E”, *Magenta* and *Orange*), showing that the orbital maximal repulsion model was on average better capturing the long distance range organization of CCs.

**Figure 6:**
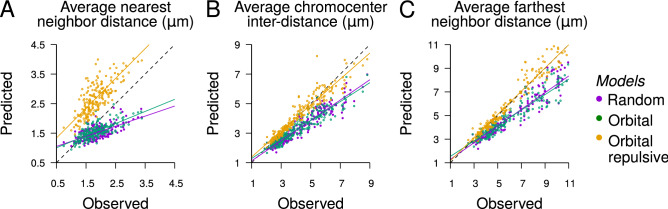
Observed and predicted average distances under the completely random (*Magenta*), the orbital (*Green*), and the orbital maximal repulsion (*Orange*) models. (**A**) Average distance between each chromocenter and its closest neighbor. (**B**) Average distance between pairs of chromocenters. (**C**) Average distance between each chromocenter and its farthest neighbor.

To assess the amplitude of differences between observed and modeled patterns, we compared average distances under the three tested spatial models. The average nearest neighbor distance confirmed the significant departure at the local scale between observed CC distributions and each of the three models (Fig. 6A; for all models, $P\mathrm{-value}<2.2~10^{-16}$, Wilcoxon signed rank test). The average inter-chromocenter distance confirmed the more repulsive organization of CCs compared with the completely random and orbital models ($P\mathrm{-value} <2.2~10^{-16}$), and the closer organization to that predicted by the orbital maximal repulsion model (Fig. 6B; $P\mathrm{-value}=0.66$). The average distance to the farthest CC was in perfect agreement between observations and predictions under the orbital maximal repulsion model (Fig. 6C; $P\mathrm{-value}=0.99$), thus confirming the scale-dependent behavior of CC organization. Overall, our results strongly suggest a multiscale organization of CCs with a maximally regular organization at the global scale and, simultaneously, a non-maximally regular organization at the local scale.

## Discussion

Because space matters in biological processes, we developed a new statistical framework to evaluate advanced spatial models that could account for biological object 3D distribution and help to understand functional correlations and identify determinants of spatial arrangements. As a study case, we explored the organizational rules underlying the nuclear distribution of the constitutive heterochromatin compartment in interphase nucleus of the *A. thaliana* plant model. With our framework, we could address new questions and reveal several new features in the spatial arrangement of *A. thaliana* chromocenters.

### An objective demonstration of chromocenter peripheral location

It is commonly accepted that CCs are located preferentially at the nuclear periphery^35,36^. Observing proximity alone is not sufficient to conclude to a significant interaction, since there is a high probability for a point to be located by pure chance close to the periphery^19^. Methods have been proposed to test the statistical significance of spatial interactions between positions and nuclear boundary^37–40^ and more generally between positions within a domain and the domain boundary^41^. Because CCs are real-size objects that cannot be assimilated to points, these methods cannot be applied to evaluate their peripheral positioning. It is well known that neglecting object size in spatial analysis can cause spurious detection of spatial interactions^42–44^. We show here that in a confined domain, randomly distributed objects are located relatively closer to the periphery when their size increases, as a result of mutual exclusion. Hence, comparing observed CCs patterns with a uniform distribution of points could lead to erroneously conclude to a significant peripheral positioning. The framework we propose avoids these pitfalls by integrating unbiased statistical tests coupled to spatial distance functions *B* and *C*. We corroborate previously reported observations^38,40^ by presenting the first objective demonstration of a peripheral positioning of CCs at the nuclear boundary. Our approach provides the basis for sound assessments of dynamics at the nuclear periphery under various physiological, genetic or environmental conditions.

Recent works have put forward the role of phase separation in the compartmentalization of chromatin and the importance of tethering at the periphery in the conventional nuclear organization^45–48^. Though physical interactions between plant heterochromatin domains and nuclear periphery have been reported^49–52^, the molecular basis of the peripheral localization of CCs remains unknown. In addition, increased distance between CCs and nuclear border have been reported in mutants of nuclear envelope components^40^, but it remains unknown whether the preferential positioning is also affected. The ability of our methodology to quantitatively assess spatial interactions with nuclear boundary will be useful to address these questions.

### New dimensions in the spatial organization of chromocenters

**Figure 7:**
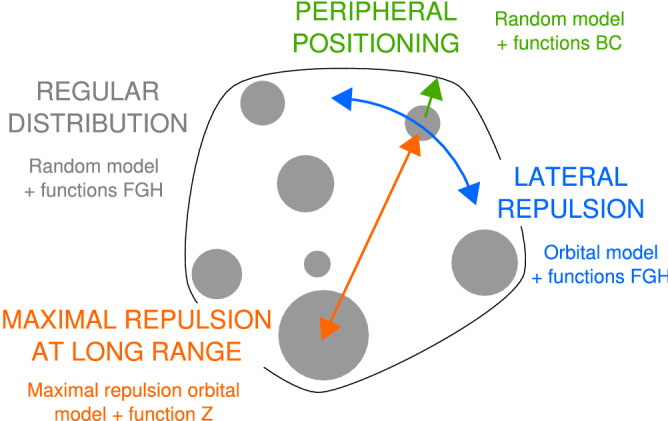
Schematic summary of elements involved in the 3D organization of heterochromatin. The three spatial models (random model, orbital model and maximal repulsion orbital model), combined with functions *B*, *C*, *F*, *G*, *H* and *Z*, unraveled new properties of *Arabidopsis* chromocenter distribution. A complex, multiscale and multiaxis organization of chromocenters was revealed using the proposed framework.

We found that the CC distribution was more repulsive than expected under the orbital model we introduced, thus showing that the peripheral positioning of CCs is not sufficient to explain their apparent repulsion. Hence, our results lead to a reassessment of the common view of the peripheral location of CCs and suggest that they organize instead along two directions in the nuclear space, one radial (peripheral positioning) and one lateral (regular positioning at the periphery). In addition, our new orbital maximal repulsion model was close to the observed distribution of CCs at the largest spatial scale. Hence, spatial interactions between CCs depend on the considered spatial scale, with close to maximally repulsive spatial interactions at long range. Overall, our study highlights complex, multi-scale spatial interactions inside the cell nucleus with new interactions between CCs beside their putative interactions with the nuclear periphery (Fig. 7).

The organization principles we revealed seem robust and shared, since they were independent from the observed diversity in nuclear morphology and number of CCs. The observed nuclear heterogeneity reflects the diversity of cell type and size present in plantlet tissues^53,54^, and also suggests variability in ploidy levels since DNA content, nuclear size, and cell size are correlated^55,56^. Some nuclei contained more than 10 CCs, confirming a significant amount of polyploidy was present in the analyzed population, given in addition that the proportion of such nuclei only provides an under-estimate of the actual level of polyploidy. This heterogeneity had no counterpart in the SDI distributions obtained under different models and spatial descriptors and we found no relation with CC spatial organization. The ploidy-independent CC organization thus suggested by our spatial analyses is consistent with previous studies reporting independence between nuclear size and morphology of CC patterns in leaf nuclei^57^, conserved spatial organization of *A. thaliana* chromosome territories between nuclei of different morphologies and cell types^58^ or ploidy level^59^, and similar HiC maps in diploid and polyploid nuclei^51^. We previously demonstrated that the non-random distribution of CCs was shared between plant and animal species^32^. Whether the new principles shown here are also more widely conserved remains to be elucidated.

Since observed patterns did not show maximal repulsion in the short distance range, the close to maximally negative interactions at long range cannot be interpreted as resulting from the propagation of repulsive short range interactions. Simple geometric constraints generated by nuclear partitioning can be invoked when questioning the mechanistic basis and functional relevance of CC regular arrangements. The partitioning of the nuclear space into CTs, which was previously hypothesized to restrain CC associations^60^, could indeed contribute to their spatial regularity. Whether the confinement of CCs to CTs would be sufficient to account for observed regularity in both the short and long distance ranges could be tested within our framework based on the simultaneous labeling of all CTs and CCs. The distribution of CCs can alternatively be interpreted as driving, rather than resulting from, the arrangement of CTs. For instance, numerical experiments showed that attachments of chromosomal regions at the nuclear envelope can impact global chromosome organization and intra- as well as inter-chromosome contacts, with CTs being more territorial and establishing less contacts with others when the number of attachments increased^61^. Given the anchoring of chromatin loops at CCs in *Arabidopsis*^36^, the regular distribution of CCs at the periphery could similarly contribute to limiting the intermingling and contacts between chromatin loops.

Beyond putative interactions with nuclear envelope and constrains imposed by CTs, several nuclear compartments and factors may also participate to CC spatial organization. For example, specific genomic regions (Nucleolus Associated Domains) including centromeric and pericentromeric regions were found associated with the nucleolus^16^, which was recently shown to subtend high-order interactions across regions from different chromosomes^62^. However, much less is known about nuclear organization in plants than in mammals and profound differences may exist between the two reigns. In *A. thaliana*, telomeres preferentially associate with the nucleolus, while centromeres and CCs are excluded from it except for chromosome 4, which bears the active NOR^35,36,63^. Our methodology is a statistical one that addresses organization principles on a population of objects. It is thus unclear to what extent the sole association of NOR4 with the nucleolus may be determinant in the spatial organization traits we reveal here, given in addition that interaction with the nucleolus should probably favor a clustered rather than a regularly dispersed distribution of CCs. Though positive spatial interactions with the nucleolus are unlikely to subtend our observations, the recently reported increased association of centromeric regions with the nucleolus in the *nuc1* nucleolin mutant^63^ raises the question of whether negative spatial interactions between CCs and the nucleolus may participate to the repulsive organization shown here. Other nuclear factors are likely involved, as suggested for example with recent studies reporting increased centromere association in mutants of plant condensin II subunits^64,65^.

### A new framework for spatial modeling of biological patterns

The spatial analysis of biological point patterns in biological imaging generally rely on single patterns, with a single spatial descriptor, and on complete spatial randomness as reference model. The proposed framework extends these limits by allowing analysis over collections of patterns, combining several classical and new descriptors, and by introducing several new models.

Testing complete spatial randomness of points or objects is generally considered as a first step for spatial analyses^5^, allowing to evaluate the existence of non-random organization rules and to reveal attractive or repulsive trends. However, the completely random model does not allow to question the origin of non-randomness. Spatial heterogeneity (non-uniform probability for an object of being located at some position) and spatial interactions (mutual dependence between object positions) are the two possible, non exclusive causes of departure from complete randomness in a confined domain. Spatial heterogeneity alone can cause spurious interactions, as highlighted in our numerical experiments where interactions with the border induced apparent interactions between objects that were actually independently positioned. In addition, interactions between objects can also superimpose to interactions between objects and domain boundary. Our framework allows to evaluate the existence and the separate contributions of different interactions, as illustrated here using the orbital model. Similarly, the amplitude of repulsion between objects could be assessed using the maximal repulsion model. Beyond the models introduced here, the framework can be extended using any spatial model that can be simulated within confined 3D domains, thus allowing to finely dissect the rules that underlie spatial organizations.

Most recent studies of biological point patterns rely exclusively on the use of Ripley’s function *K*, a spatial descriptor closely related to function *H*. However, function *K* captures only part of the spatial information and different processes can exhibit the same function *K*^66^. In line with other authors^5,67^, we advocate using a combination of spatial descriptors to test a model on a data set. One drawback is the need to perform multiple goodness-of-fit tests with different descriptors, which may lead to incorrect type-I error if correction methods^68,69^ are not considered. In the present work, the strong departures between most observed SDI distributions and uniformity provide sufficient confidence that our conclusions were not simply due to multiple testing. In addition, this potential drawback is moderate compared with the benefits. Combining new distance functions (*B*, *C*, and *Z*) with classical ones (*F*, *G*, and *H*) was essential to reveal the multiple dimensions of CC spatial organization (Fig. 7). First, the objective demonstration of CC peripheral positioning could not have been achieved without combining functions *B* and *C*, because *B* was not sensitive to the peripheral positioning in flat nuclei. It has been proposed that nucleus flattening should be taken into account by performing analysis in 2D instead of 3D^70^. We believe that combining complementary descriptors is a more suitable alternative, in particular when analyzing heterogeneous samples, as it fully exploits the available 3D image information. Second, while functions *G* and *Z* capture information also quantified by function *H*, the benefit of evaluating models using each of these descriptors is to focus on different spatial scales. Since the SDI only captures the largest deviation from the model, using multiple descriptors is useful when there are different, possibly antagonist effects depending on the spatial scale.

The expansion of techniques that provide unprecedented spatial information, such as single molecule localization microscopy, and the concomitant recognition of spatial patterning as a key feature of biological systems makes statistical methods for assessing spatial models essential in biological image analysis. In the current era of high-throughput data acquisition and analysis, we anticipate increasing needs for analyzing large collections of object patterns from subcellular to tissular scales. Because it accommodates for sample heterogeneity with respect to domain geometry and object numbers and sizes, we believe our generic framework represents a useful contribution for future large-scale analyses of biological spatial patterns.

## Methods

### Synthetic spatial data

Patterns of 10 spherical objects each were simulated within a spherical domain of radius 30 voxels. Object radius was the same for all objects and set to 5 voxels. Patterns were simulated according to the completely random model of object distribution (see below), or with attraction to the border of the domain, or with repulsion from the border of the domain. Each pattern was simulated by introducing the objects one after the other. For each new object, a valid candidate position (i.e., a position for which no intersection with the border or with previously positioned objects occurred) was selected uniformly at random within the spherical domain. For the model with attraction to the border, the position was accepted if its distance *d* to the border was smaller than a fixed distance $\mu$. Otherwise, the position was accepted with a probability that decreased when the distance to the border increased:

$$\begin{aligned} \mathrm {Prob} = \exp \left( -\frac{1}{2}\left( \frac{d-\mu }{\sigma }\right) ^2\right) \end{aligned}$$

In case the position was not retained, a new candidate position was selected. For the model with repulsion from the border, the procedure was symmetrical: the position was retained with the above probability if closer than $\mu$ from the border, and systematically accepted otherwise. We set $\mu$ to 5 voxels for the border attraction model and to 15 voxels for the border repulsion model. For both models, we set $\sigma$ to 5 voxels. For the completely random model, the candidate position was systematically accepted. For each of the three spatial models, we generated 100 images of simulated patterns.

### Biological material

Seeds of Columbia (Col-0) *A. thaliana* accession were sown on sterile medium^71^ and grown *in vitro* at $20\,^{\circ }\hbox {C}$, 70% humidity, $36~\upmu \hbox {mol}\,\hbox {m}^{-2}\,\hbox {s}^{-1}$ light intensity and under short day conditions (8 h light/16 h dark). Young seedling (18-day-post-germination) were harvested, fixed in 4% paraformaldehyde in 1 $\times$ PBS buffer and used to prepare a nuclei suspension as described in^32^. The fixed plantlets were chopped in 500 $\upmu$l of extraction buffer (10 mM Tris–HCl pH 7, 4 mM spermidine, 1 mM spermine, 5 mM MgCl$_2$, 0.1% triton X-100, 5 mM $\beta$-mercaptoethanol). After filtration through a 50 $\upmu$m nylon mesh, the suspension was centrifugated (5000*g*, 3 min) and the pellet washed in 1 $\times$ PBS, treated with 0.5% triton X-100 in 1 $\times$ PBS and washed in 1 $\times$ PBS. Nuclei were resuspended in 30 $\upmu$l 1 $\times$ PBS. An aliquot was spotted on a slide, left to evaporate at $4\,^{\circ }\hbox {C}$ for 20 min. Counterstaining was performed to visualize the CCs independently from their chromosomal origin by mounting samples in VECTASHIELD antifade mounting medium (Vector laboratories) with 1 mg/ml of DAPI. A total of 210 nuclei were analyzed.

### Nucleus imaging

Images of isolated nuclei were acquired on a Leica SP2 AOBS or a LSM ZEISS 710 confocal microscope both equipped with a 405 nm diode and with a 63 $\times$ HCX PL APO (NA 1.2, WD 220 $\upmu$m) and a 63 $\times$ Plan-APOCHROMAT (NA 1.4, WD 190 $\upmu$m) objectives, respectively. The 3D image stacks were acquired with a voxel size of 0.05–0.01 $\upmu$m in the XY plane and of 0.01–0.02 $\upmu$m along the Z-axis. The anisotropy of voxel sizes in XY-Z was taken into account in all subsequent size, shape, and distances measurements.

### Image processing and analysis

Nuclei and chromocenters were segmented from 3D DAPI images (Fig. 1A) as detailed previously^32^. In short, binary masks of nuclei were obtained by filtering then thresholding images using Otsu’s method followed by threshold correction. Morphological operators were applied to fill holes and to regularize nuclear contours (Fig. 1B). A Marching Cubes algorithm was applied to the binary mask of each nucleus to generate a triangular mesh of the nuclear boundary (Fig. 1D, *Grey mesh*). Segmented images of chromocenters were obtained using the watershed transform run on the Gaussian gradient of the intensity image, followed by morphological operators on region adjacency graphs and thresholding on region constrast (Fig. 1C). The centroid and equivalent spherical radius of each chromocenter were computed and used to represent the chromocenter by its equivalent sphere (Fig. 1D, *Color spheres*). Detailed procedures and algorithms are given in^72^.

### Distance distribution functions

Object patterns were quantitatively characterized using classical (*F*, *G*, and *H*;^5^) and new (*B*, *C*, and *Z*) distance distribution functions. We note ${\mathcal {P}}$ a pattern of *N* objects represented by their centroid positions $p_1,\ldots ,p_N$ within a finite domain *D*.

Function *F* is the cumulative distribution function (CDF) of the distance between any point position $q\in D$ and the centroid of the closest object (Fig. 2B):

1 $$\begin{aligned} F(x) = P(\Vert q-\eta (q)\Vert <x) \end{aligned}$$

where $\eta (q)=\arg \min _{p\in {\mathcal {P}}}\Vert p-q\Vert$. Function *F* quantifies the void spaces between objects. Large spaces are expected in the case of object aggregation, while smaller and more regular spaces are expected in the case of a repulsive-like distribution.

*F* was estimated by first defining a set of *L* positions $\{q_1,q_2,\ldots ,q_L\}$ inside the nucleus and by then computing the distances to their nearest chromocenters. The estimation ${\hat{F}}(x)$ of *F*(*x*) was the observed proportion of distances inferior to *x*:

2 $$\begin{aligned} {\hat{F}}(x)=\frac{1}{L}\sum _{k=1}^L {\mathbb{1}}_{\{\Vert q_k-\eta (q_k)\Vert <x\}} \end{aligned}$$

where ${\mathbb{1}}_{\{E\}}$ is the indicator function of event *E*. The positions $q_1,\ldots ,q_L$ were taken as the set of voxel centers in the nucleus binary mask.

Function *G* is the CDF of the distance between any object *p* and its nearest neighbor (Fig. 2C):

3 $$\begin{aligned} G(x) = P(\Vert p-\eta (p)\Vert <x) \end{aligned}$$

Small (large) distances to the nearest neighbor are expected in the case of attraction (repulsion) between objects.

Function *G* was estimated using the empirical distribution of this distance:

4 $$\begin{aligned} {\hat{G}}(x)=\frac{1}{N}\sum _{i=1}^N {\mathbb{1}}_{\{\Vert p_i-\eta (p_i)\Vert <x\}} \end{aligned}$$

Function *H* is the CDF of the distance between any two object centroids $p_i$ and $p_j$ (Fig. 2D):

5 $$\begin{aligned} H(x) = P(\Vert p_i-p_j\Vert <x) \end{aligned}$$

Function *H* captures the spatial correlations between objects depending on their distances.

Function *H* was estimated from the empirical distribution:

6 $$\begin{aligned} {\hat{H}}(x)=\frac{2}{N(N-1)}\sum _{i=1}^N\sum _{j=i+1}^N {\mathbb{1}}_{\{\Vert p_i-p_j\Vert <x\}} \end{aligned}$$

Function *B* is the CDF of the Euclidean distance between the centroid of any object and the closest point on the border $\partial D$ of the domain (Fig. 3B):

7 $$\begin{aligned} B(x) = P(\Vert p-p'\Vert <x) \end{aligned}$$

where $p'=\arg \min _{q\in \partial D}\Vert p-q\Vert$. This distance should be smaller than under randomness if objects are preferentially located close to the border, and larger if objects tend to avoid peripheral locations (Figure S1D and F).

Function *B* was estimated using:

8 $$\begin{aligned} {\hat{B}}(x) = \frac{1}{N}\sum _{i=1}^N{\mathbb{1}}_{\{\Vert p_i-p_i'\Vert < x\}} \end{aligned}$$

where $p_i'$ is the closest point to $p_i$ on the observed boundary $\partial {\hat{D}}$ (Fig. 3A).

Function *C* is the CDF of the distance between the centroid of any object and the centroid *M*(*D*) of the domain (Fig. 3C):

9 $$\begin{aligned} C(x)=P(\Vert p-M(D)\Vert <x) \end{aligned}$$

This distance should be larger than under randomness if objects are preferentially located close to the border, and smaller if objects tend to avoid peripheral positions.

Function *C* was estimated using:

10 $$\begin{aligned} {\hat{C}}(x) = \frac{1}{N}\sum _{i=1}^N{\mathbb{1}}_{\{\Vert p_i-M({\hat{D}})\Vert < x\}} \end{aligned}$$

where $M({\hat{D}})$ is the centroid of the observed domain.

Function *Z* is the CDF of the distance between the centroid of any object and the centroid of its farthest neighbour (Fig. 5E):

11 $$\begin{aligned} Z(x) = P(\Vert p-\eta ^*(p)\Vert <x) \end{aligned}$$

where $\eta ^*(p)=\arg \max _{q\in {\mathcal {P}}}\Vert p-q\Vert$. Large values of this distance are expected if there are negative interactions between object positions in the long distance range. Conversely, smaller values than under complete randomness are expected if objects form a single cluster.

Function *Z* was estimated as:

12 $$\begin{aligned} {\hat{Z}}(x)=\frac{1}{N}\sum _{i=1}^N {\mathbb{1}}_{\{\Vert p_i-\eta ^*(p_i)\Vert <x\}} \end{aligned}$$

### Completely random model of object distribution

The completely random model of object distribution in a bounded domain of the 3D space was defined as a model wherein each object is uniformly distributed in the domain and where the positions of different objects are independent, under the constraint that objects cannot intersect and cannot intersect with the domain boundary. The input parameters of the model are the domain boundary, the number of objects, and the size (radius) of each object.

We designed an algorithm for generating completely random object patterns, in which objects are added one after the other, taking care not to intersect previously placed objects and domain boundary. Noting $p_i$ and $r_i$ the position and radius of object *i*, and $p_i'$ its closest point on domain boundary $\partial D$, these conditions translate respectively to $\Vert p_i-p_j\Vert \ge r_i+r_j$ and $\Vert p_i-p_i'\Vert \ge r_i$ (Fig. 2A). Depending on the position of already placed objects, it can happen that the next object cannot be placed without causing an intersection. We incorporated two mechanisms to prevent the algorithm from being stuck in such a configuration: (i) when the number of failed attempts to place an additional object had reached a threshold, the process was restarted from the first object; (ii) the order of introduction of the objects into the algorithm was randomized before placing the first object.

### Orbital model

The algorithm we designed for generating object patterns according to the orbital model was similar to the algorithm used for complete randomness, with the additional constraint that the centroid of each object was constrained to remain on its orbit (Fig. 4A). The additional input parameters in the orbital model, compared to the completely random model, were the distances to the domain boundary that defined the individual orbits of the objects.

### Maximal repulsion models

Algorithms are available for simulating classical repulsion models such as Matérn’s Poisson hard core processes and Gibbs point processes^73,74^. A procedure for simulating repulsive patterns was also proposed in^75^ based on simulating a number of completely random patterns and selecting the one with maximum average nearest neighbor distances. These models and algorithms, however, do not guarantee that maximal repulsion and regularity are achieved. We therefore designed an algorithm to simulate patterns with maximal repulsion. The algorithm minimizes an energy function defined on the space of possible object configurations. The energy of any object configuration, corresponding to a set of object centroid positions $p_1,\ldots ,p_N$, was defined as the opposite average distance from each object to its nearest neighbor:

13 $$\begin{aligned} E(p_1,\ldots ,p_N) = -\frac{1}{N}\sum _{i=1}^N \min _{j\not =i}\Vert p_i-p_j\Vert \end{aligned}$$

Drawing a realization of the maximal repulsion model started from a completely random object configuration. The Metropolis algorithm^76^ was then used to iteratively converge towards a configuration minimizing *E*. At each step, one object was picked up at random and randomly moved within the neighborhood of its current position, avoiding intersection with domain boundary and with other objects. The energy difference $\Delta E$ corresponding to this configuration change was computed. The move was systematically accepted if $\Delta E < 0$. Otherwise, the move was accepted with probability $\exp (-\beta \Delta E)$. The procedure was repeated until convergence, as defined by the stabilization of energy.

The orbital maximal repulsion model was obtained by combining the orbital model and the maximal repulsion model. The algorithm for simulating a realization of this model started with a realization of the orbital model and then applied the energy minimization procedure used for the maximal repulsion model, with the additional constraint that the distances between object centroids and domain boundary were kept constant.

### Statistical tests

For each input pattern (synthetic or experimental) and each distance function (*F*, *G*, etc.), the estimate of the function expected under the model (e.g., ${\hat{F}}_{\mathrm {rand}}$, ${\hat{G}}_{\mathrm {rand}}$, etc. for the completely random model) was computed by averaging over 99 model simulations. For all models, the domain boundary, the number of objects and the object sizes used in simulations were taken from the input pattern. For the orbital model and the maximally repulsive orbital model, the orbital distances were set to their values measured in the input pattern. The maximal signed vertical distance *S* between the observed pattern function and the model function was measured. For function *F*, for example:

$$\begin{aligned} S=F(x^*)-{\hat{F}}_{\bullet }(x^*) \end{aligned}$$

where $x^*$ is given by:

$$\begin{aligned} x^*=\arg \max _{x\ge 0}|F(x)-{\hat{F}}_{\bullet }(x)|. \end{aligned}$$

A second set of 99 simulations was computed. The Spatial Distribution Index (SDI) was defined as the proportion of these simulations for which *S* was below the measured one.

The uniformity of SDI distributions was tested by applying the two-sided Kolmogorov-Smirnov test under the R software version 3.4.4^77^. For all SDI distributions reported from Figs. 2, 3, 4 and 5, the *P* value of the uniformity test was below machine precision.

Observed and model-predicted average distances were compared using Wilcoxon signed rank test for matched samples in the R software.

## Supplementary Information

Supplementary Figures.

## Data Availability

The data set of 3D nuclear images generated and analyzed in this study has been deposited in the Data INRAE portal and is publicly available at https://doi.org/10.15454/1HSOIE.

## Code availability

The C++ source code of the program for simulating synthetic patterns, quantifying segmented images of 3D patterns and for evaluating spatial models has been deposited on the Data INRAE portal and is available at https://doi.org/10.15454/UCRGED. A compiled version of the program running on Ubuntu 18.04 is also included.
