# Publisher Correction: Spatial modeling of biological patterns shows multiscale organization of *Arabidopsis thaliana* heterochromatin

**DOI:** 10.1038/s41598-021-87458-7

**Published:** 2021-05-11

**Authors:** Javier Arpòn, Kaori Sakai, Valérie Gaudin, Philippe Andrey

**Affiliations:** Institut Jean-Pierre Bourgin, INRAE, AgroParisTech, Université Paris-Saclay, 78000 Versailles, France

Correction to: *Scientific Reports* 10.1038/s41598-020-79158-5, published online 11 January 2021

The original PDF version of this Article contained a repeated error where the symbol “ ” was incorrectly given as “ ” in the Methods section, under the subheading ‘Distance distribution functions’ and in Equations 2, 4, 6, 8, 10 and 12.

This error has now been corrected in the PDF version of the Article; the HTML version was correct from the time of publication.

