# Supplementary material for: Spatial modeling of biological patterns shows multiscale organization of Arabidopsis thaliana heterochromatin
Source: Sci Rep. 2021 Jan 11;11:323. doi: 10.1038/s41598-020-79158-5 (PMC7801681; doi:10.1038/s41598-020-79158-5)
Supplement: Supplementary file 1 — Supplementary Figures. [file 41598_2020_79158_MOESM1_ESM.pdf]

Spatial modeling of biological patterns shows multi-scale organization of ***Arabidopsis thaliana*** heterochromatin

Javier Arpòn[1], Kaori Sakai[1], Valérie Gaudin[1,*], Philippe Andrey[1,*]

[1]Institut Jean-Pierre Bourgin, INRAE, AgroParisTech, Université Paris-Saclay, 78000 Versailles, France

* To whom correspondence should be addressed.

E-mail: valerie.gaudin@inrae.fr, philippe.andrey@inrae.fr

Supplementary Information

Supplementary Figure S1: Statistical spatial analysis of synthetic patterns.

Supplementary Figure S2: F-SDIs computed with the completely random model are not related to nucleus morphology or ploidy-related parameters.

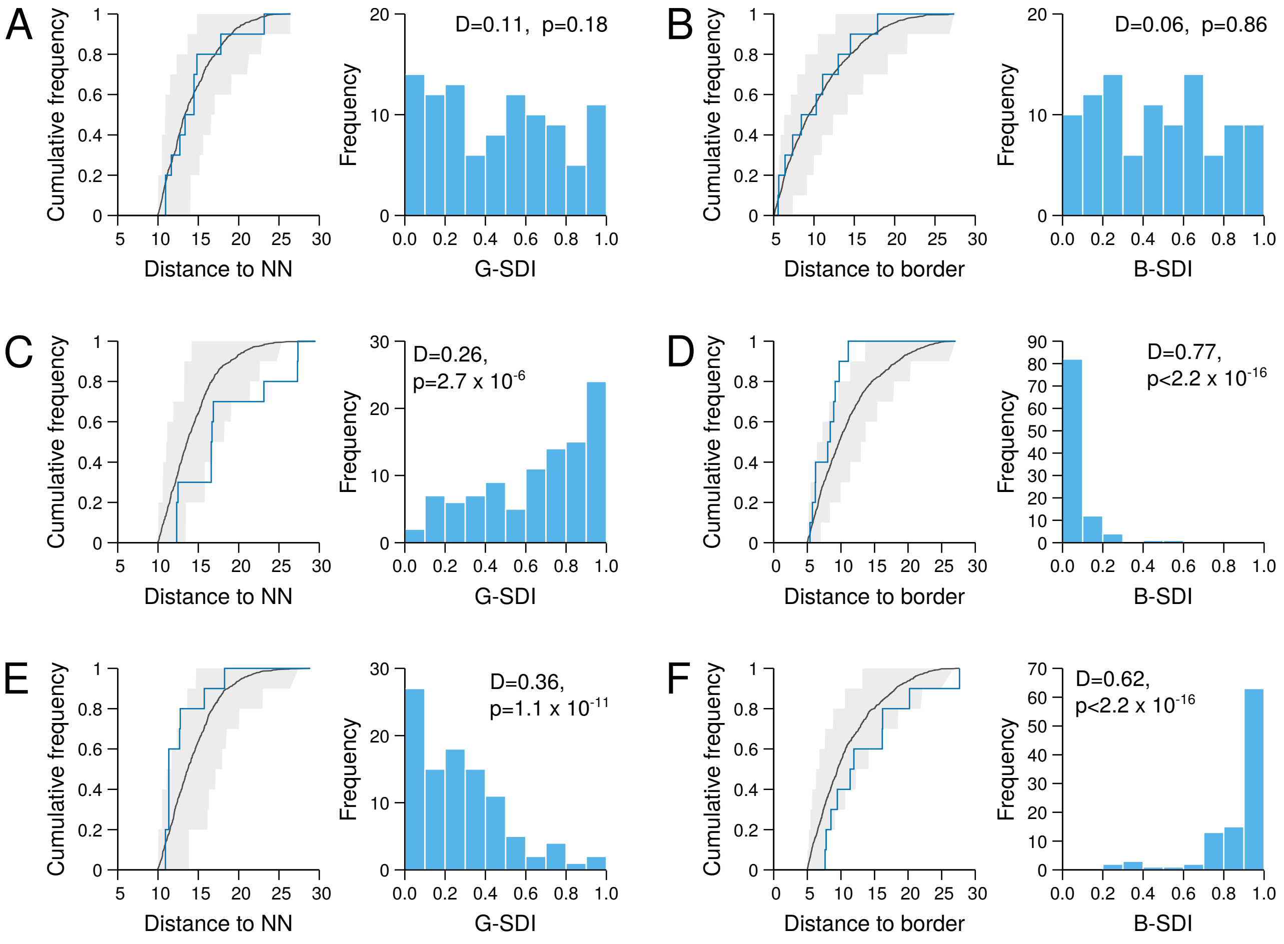

Supplementary Figure S1: Statistical spatial analysis of synthetic patterns. Three sets of synthetic patterns were generated according to three types of spatial distribution rules and compared to the completely random model of object distribution (AB, CD, EF). For each type, 100 patterns of 10 objects each were simulated within a sphere. (AB) Patterns simulated according to the completely random model. (A) *Left*: Individual function G (*Blue*), function G expected under the completely random model (*Dark grey*), and 95% envelope expected under the completely random model; *Right*: population distribution of the G-SDI. NN: nearest neighbor. (B) Individual function B (*Left*) and population distribution of the B-SDI (*Right*). In (AB), note how uniform SDI distributions are obtained, highlighting the adequacy of the completely random model with the analyzed patterns. (CD) Same as (AB) for patterns simulated with attraction of objects to the border. (EF) Same as (AB) for patterns simulated with repulsion of objects from the border. In (CD) and (EF), note the departure from uniformity of the SDI distributions, as expected from the non-completely random spatial distribution rules. D and p: statistics and p-value, respectively, of the Kolmogorov-Smirnov test of uniformity of the SDI distribution ($n = 100$).

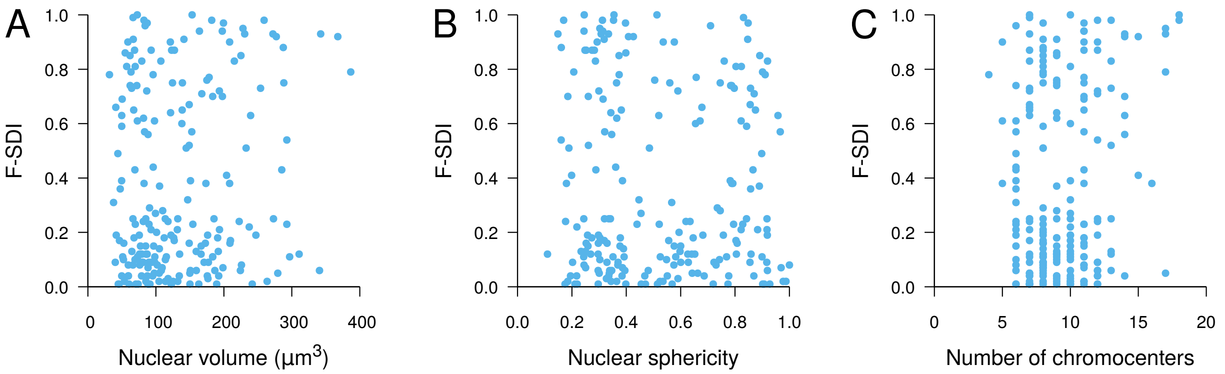

Supplementary Figure S2: F-SDIs computed with the completely random model are not related to nucleus morphology or ploidy-related parameters. The F-SDIs obtained by comparing observed CC patterns to patterns predicted by the completely random model are shown as a function of nuclear volume (A), nuclear sphericity (B) and number of chromocenters (C). The existence of a relation between the F-SDI and each of these parameters was statistically evaluated by splitting the nuclei in two groups with F-SDI below and above 0.5, respectively, and by comparing average parameter values between the two groups. There was no significant difference at the 5% significance level (Wilcoxon test, $N = 210$; nuclear volume: $W = 5345$, p-value $= 0.31$; nuclear sphericity: $W = 4671$, p-value $= 0.54$; number of chromocenters: $W = 5541$, p-value $= 0.14$).
